# Supplementary material for: A tale of two distinct actin networks underlies the entire life cycle of focal adhesion
Source: bioRxiv. 2025 Nov 13:2025.11.12.688129. Preprint. [Version 1] doi: 10.1101/2025.11.12.688129 (PMC12642665; doi:10.1101/2025.11.12.688129)
Supplement: 1 [file NIHPP2025.11.12.688129v1-supplement-1.pdf]

bioRxiv preprint doi: https://doi.org/10.1101/2025.11.12.688129; this version posted November 13, 2025. The copyright holder has placed this preprint (which was not certified by peer review) in the Public Domain. It is no longer restricted by copyright. Anyone can legally share, reuse, remix, or adapt this material for any purpose without crediting the original authors.

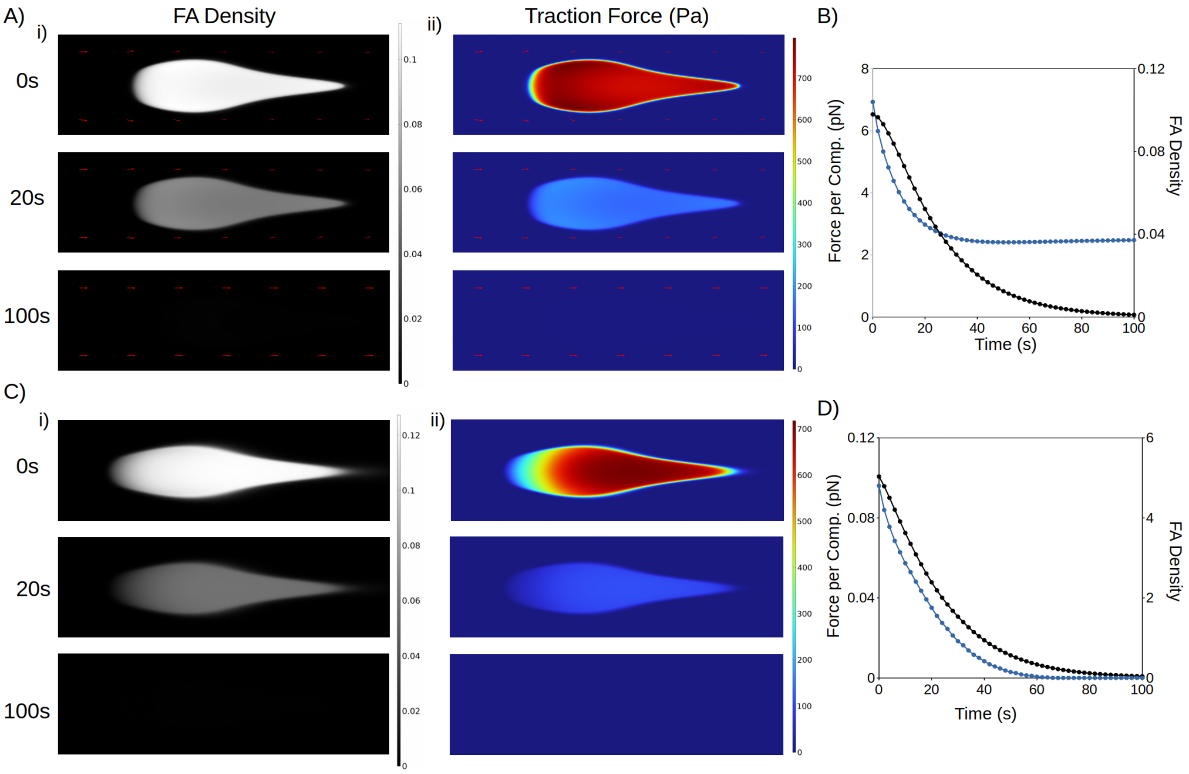

Figure S1: Substantial perturbations to contractility and the overlying actin network can drive FA disassembly. (Ai, ii) FA Density (Ai) and traction in Pascals (Aii) after reducing actomyosin contractility on-rate at the FA to zero. In the absence of actomyosin contractility, the rear of the FA becomes mechanically disengaged, leading to its disassembly. (B) Force per component and FA Density over 100s after setting contractility on-rate to zero. Force per component is in blue and FA density is in black. (Ci, ii) FA Density (Ci) and traction in Pascals (Cii) after reducing both retrograde actin flux and contractility on-rate to zero. With no source of mechanical engagement, catch-bonds become completely disengaged, leading to the disassembly of the entire FA. (D) Force per component and FA density over 100s after setting both flux and contractility on-rate to zero. Force per component is in blue and FA density is in black.

bioRxiv preprint doi: https://doi.org/10.1101/2025.11.12.688129; this version posted November 13, 2025. The copyright holder has placed this preprint (which was not certified by peer review) in the Public Domain. It is no longer restricted by copyright. Anyone can legally share, reuse, remix, or adapt this material for any purpose without crediting the original authors.

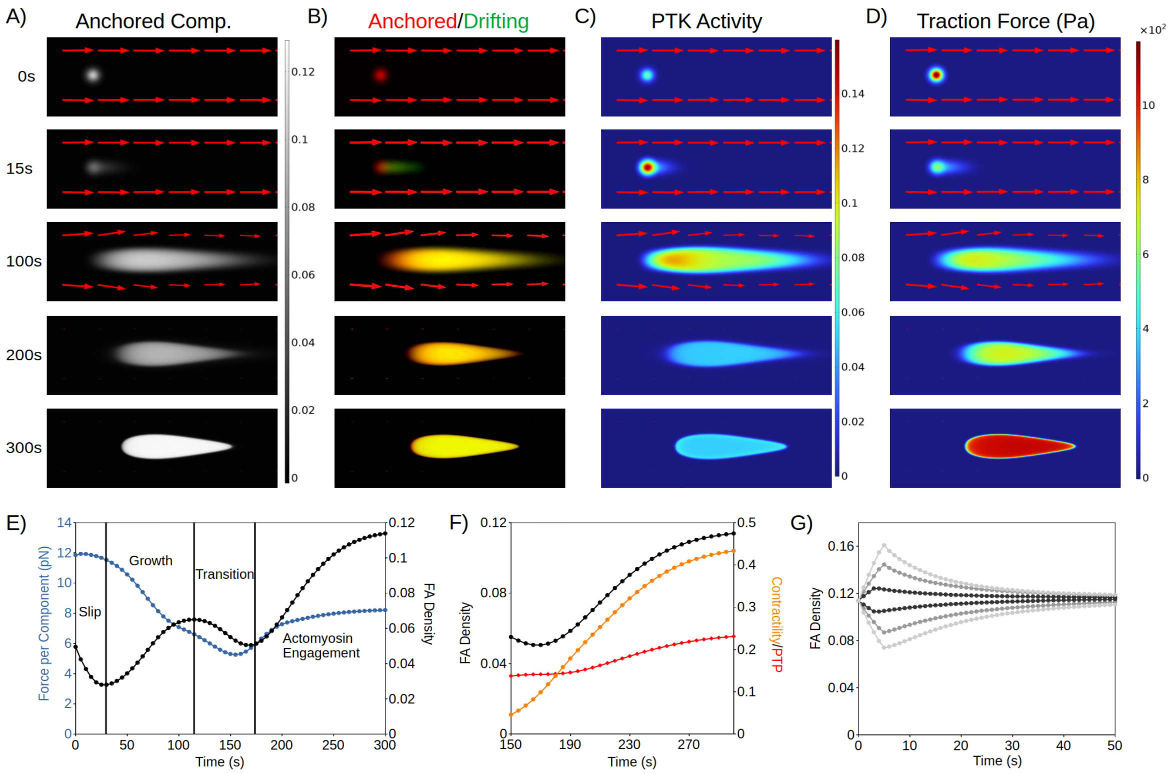

Figure S2: Inclusion of myosin load-dependence sustains key model predictions on FA assembly and stability. (A) Distribution of anchored FA components over 300s. Arrows indicate retrograde actin flux; length of arrows indicate flux magnitude. (B) Anchored and drifting FA components over 300s. Anchored components are in red, while drifting components are in green. (C) PTK Activity over 300s. The prediction that PTK is high during initial assembly and forms a gradient towards the distal end is preserved. (D). FA traction force over 300s. (E) FA Density and Force per Component (pN) over 300s. FA Density is in black, while Force per Component is in blue. The FA initially undergoes slipping upon engagement with actin, before forming a stronghold and growing. The FA then enters the lamella at 120s (modeled as a reduction of retrograde flux to 5nm/s), at which point the FA is sustained by actomyosin contractility. (F) Contractility, PTP activity and FA density from 150s to 300s after the start of the simulation. Contractility is in orange, PTP activity is in red, and FA density is in black. (G) FA Density in response to perturbations of different amplitude (0.05-0.15 1/s) exerted over 5s. The FA maintains stability in the face of small perturbations, as previously predicted.

bioRxiv preprint doi: https://doi.org/10.1101/2025.11.12.688129; this version posted November 13, 2025. The copyright holder has placed this preprint (which was not certified by peer review) in the Public Domain. It is no longer restricted by copyright. Anyone can legally share, reuse, remix, or adapt this material for any purpose without crediting the original authors.

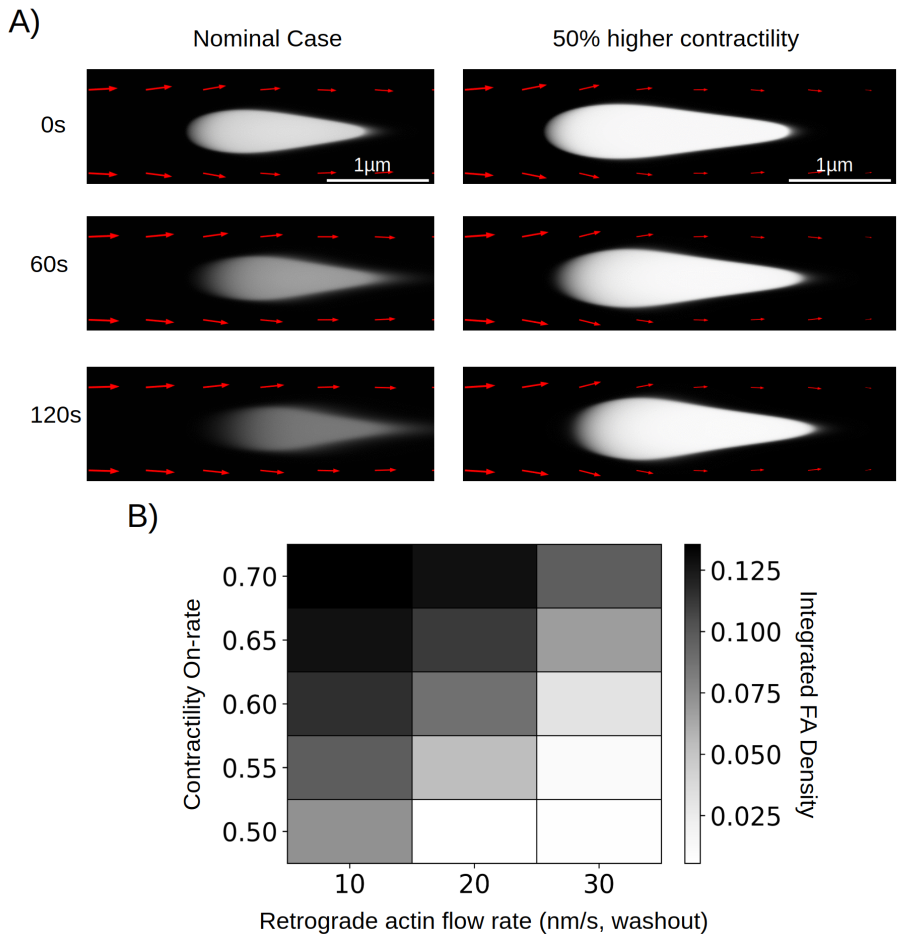

Figure S3: Increasing contractility in tandem with retrograde actin flow leads to FA growth. (A) Distribution of FA Density over 180s in the nominal case (parameters identical to Figure 5) and with 50% higher contractility (on-rate increased by 50%), mimicking the effect of global microtubule disassembly. (B) Phase plot of integrated FA density after 340s, at varying contractility on-rates (1/s) and retrograde actin flow rates after maturation in the lamella (nm/s).
